# The Burden of Hypertension in HIV-Infected Adults on Retroviral Therapy in Masvingo Province, Zimbabwe: A Retrospective Cohort Study

**DOI:** 10.7759/cureus.46476

**Published:** 2023-10-04

**Authors:** Kudzai Masunda, Zahid Khan

**Affiliations:** 1 Medicine, JF Kapnek Health Department, Harare, ZWE; 2 Acute Medicine, Mid and South Essex NHS Foundation Trust, Southend on Sea, GBR; 3 Cardiology, Barts Heart Centre UK, London, GBR; 4 Cardiology and General Medicine, Barking, Havering and Redbridge University Hospitals NHS Trust, London, GBR; 5 Cardiology, Royal Free Hospital, London, GBR

**Keywords:** hiv/aids-related morbidity and mortality, global public health, who- world health organization, anti-retroviral therapy (art), non-communicable diseases epidemiology public health community-based study, adults living with hiv, systolic blood pressure as well as diastolic blood pressure, logistic regression model, hypertension among hiv positive clients, hiv on art care

## Abstract

Background and objective

The global HIV epidemic has evolved in the past 30 years with a decline in mortality and morbidity and improved survival since the introduction of antiretroviral therapy (ART). However, this has brought on new challenges through the emergence of non-communicable disease (NCD) as a pandemic at par with, if not more serious than, HIV, and patients well maintained on ART are now faced with the increased risk of developing NCDs such as hypertension, which also require lifelong therapy. This study was designed to determine the burden of hypertension in patients under HIV care in Masvingo province, Zimbabwe.

Methods

A retrospective cohort study was conducted in six districts of Masvingo province based on the data collected from the electronic Patient Monitoring System (ePMS), along with an analysis of secondary data. Of the 94,821 records gathered, 877 met the inclusion criteria to be included in the study. Data were analyzed using Microsoft Excel and Stata statistical software and statistical analysis was performed using the χ2 test.

Results

The study revealed a hypertension prevalence of 7.64% among the 877 patients analyzed and the independent risk factors for the development of hypertension were determined to be the age of patients, with a one-year increase in age resulting in an 8% increase in the risk of developing hypertension, and the duration on ART, with a one-year increase on ART duration increasing the risk of hypertension by 27%, and an increase in BMI by a factor of 1 increasing the risk of getting hypertension by 9%.

Conclusion

Our findings showed that there are patients who have both hypertension and HIV on ART care, and they would need to be managed for both. There is a need for improved data collection of important variables that improve the quality of care of clients. Clinical management of HIV needs to evolve with the evolving needs of patients and NCD care has to be factored in.

## Introduction

HIV remains a major global public health problem. According to the World Health Organization (WHO), an estimated 36.7 million adults are living with HIV while 1.3 million acquired it in 2021 alone globally [1]. In Zimbabwe, the prevalence of HIV in adults is 11.6%. Of note, 96% (1,243,557) of adults living with HIV (ALWHIV) are aware of their status; of these, 91% (1,88,636) were receiving antiretroviral therapy (ART) in 2021 [2]. The introduction of ART has resulted in significant viral suppression and prolonged life among ALWHIV. This reduction in HIV/AIDS-related morbidity and mortality has highlighted the effectiveness of ART in improving the survival rates of ALWHIV [3].

A concerning prevalence of non-communicable diseases (NCDs), such as diabetes mellitus, cardiovascular diseases, and hypertension among ALWHIV has been reported in several studies [4-7]. While ART benefits ALWHIV by suppressing viral load, decreasing opportunistic infections, and increasing life expectancy, it can lead to various adverse effects depending on the type of medication and duration that patients have been on it [8]. Adverse effects resulting from chronic use of ART include combined phenotype of insulin resistance, visceral fat accumulation, dyslipidemia, and anemia, and the effects vary based on the class of medication [9]. Examples of adverse effects related to classes of ART have been documented, and integrase strand transfer inhibitors (INSTIs) have been associated with generalized weight gain and abdominal obesity while protease inhibitors (PIs)have been reported to cause anemia in post-partum women; however, the biomechanism of the adverse reactions to ART are poorly understood, and these reactions predispose HIV patients to hypertension [10].

Hypertension is defined by WHO as an elevated systolic blood pressure ≥140 mmHg and diastolic blood pressure ≥ 90 mmHg, and it is a major cause of premature death [11]. WHO estimates that almost half of adults with hypertension are not aware of their condition, and the organization targets to reduce the prevalence of hypertension by 33% by the year 2030 [11]. The global prevalence of hypertension has been estimated at 23.6% (95% CI: 21.6-25.5), and the prevalence varies depending on the region. The prevalence in Eastern and Southern Africa has been reported to be 19.9% (95% CI: 17.2-22.8). People living with HIV are at an increased risk of developing hypertension due to the infection itself or the effects of ART [12].

This study aimed to determine the burden of hypertension and its known risk factors like BMI, diabetes mellitus, renal and liver diseases, and the ART regimen in ALWHIV on ART and which of the risk factors contribute to the burden of hypertension. The primary objective was to determine the burden of hypertension in HIV-infected adults on ART in Masvingo province, Zimbabwe. The secondary objectives were to determine the prevalence of Hypertension in HIV-infected adults on ART, to describe the demographic characteristics of the HIV-infected adults on ART, and to analyze the factors (correlates) that predispose HIV-infected adults to hypertension.

## Materials and methods

Ethical approval and study setting

We obtained approval from the Ethical Board of the Provincial Medical Directorate for Masvingo province. This study utilized secondary data and a retrospective cohort analysis was carried out. The setting was Masvingo province, which has 165 medical facilities that offer ART in the public health system.

Study design

The study involved a retrospective cohort analysis of data collected in the electronic Patient Monitoring System (ePMS). The sampling frame was all adults in the ePMS receiving HIV care and treatment in Masvingo province’s six districts, which totaled 96,821 individuals. The study population comprised adults in Masvingo province diagnosed with HIV and who started ART between the 1st of January 2014 and the 31st of December 2022 and whose treatment data including blood pressure values were recorded in ePMS. The resultant sample after excluding all records without BP values amounted to 877 records. The inclusion and exclusion criteria are detailed below:

Inclusion criteria

The inclusion criteria were as follows: all HIV-positive patients who have commenced treatment and are at least 18 years of age, ART patients currently receiving active treatment, and patients with data including BP values and duration on ART recorded in the ePMS database.

Exclusion criteria

The exclusion criteria were as follows: ART patients below the age of 18 years, HIV-positive patients who have not started ART treatment, and patients whose data including BP values and duration on ART were not recorded in the ePMS database.

As shown in Table 1, ePMS had 99,015 records of patients from 2004 to 2022 in Masvingo province's six districts. Of these, 94,821 met the criteria related to age; 7,129 records had BP values available in the system, 1,297 had anthropometric measurements of weight and height, and of these, 877 also had data on the duration of ART, which was an important variable in the study.

**Table 1:** Summary of analyzed records ART: antiretroviral therapy; BP: blood pressure; ePMS: electronic Patient Monitoring System

Summary	
All records in ePMS for the review period	99,015
Records of patients aged 18+ years	94,821
Records with BP values	7,129
Records with BP values + anthropometric measurements	1,297
Records with BP values + anthropometric measurements and duration on ART	877

Data management and analysis

Data were retrieved from ePMS and refined using Excel and STATA. Available case analyses were used to account for the missing data. The major variables that were used in analyses were time spent on ART and hypertension status. Any record that did not have these two was not used in the analyses. Categorical variables were compared by using the χ2 test. Multiple logistic regression analysis was performed to enable the researcher to identify the significant covariates that had a bearing on explaining the association of hypertension among people on ART.

Effect modification was checked for all the covariates that were used in the model since the model had a few variables of interest that the researcher had obtained from the ePMS database. This was done by including product terms of each combination of the variables present in the logistic regression model. Upon variable selection (by backward selection method), marital status was dropped and hence not included in the final logistic regression model and subsequently not tested for effect modification. No effect modifiers were observed, and hence the model was reduced to once without any product terms. Descriptive analyses for main study variables, CHI square tests, and logistic regression analysis were carried out by using STATA.

## Results

A total of 877 records were included in the analysis. The descriptive statistics of patients on active ART treatment based on covariates are presented in Table 2. Further description of numerical variables is provided in Table 3.

**Table 2:** Descriptive statistics of clinical and demographic characteristics of patients on ART treatment ART: antiretroviral therapy: BMI: body mass index

Variable	Number	%	Cumulative %
District			
Chiredzi	232	26.45	26.45
Chivi	170	19.38	45.84
Gutu	86	9.81	55.64
Masvingo	292	33.3	88.94
Mwenezi	36	4.1	93.04
Zaka	61	6.96	100
Age group, years			
18-25	75	8.55	8.55
26-35	222	25.31	33.87
36-45	328	37.4	71.27
46-55	184	20.98	92.25
56/64	42	4.79	97.04
65+	26	2.96	100
Sex			
Female	554	63.17	63.17
Male	323	36.83	100
BMI category			
Underweight	60	6.84	6.84
Healthy weight (normal)	478	54.5	61.35
Overweight	263	29.99	91.33
Obese	76	8.67	100
Marital status			
Divorced	126	14.37	14.37
Married	576	65.68	80.05
Single	83	9.46	89.51
Widowed	92	10.49	100
Hypertensive			
No	810	92.36	92.36
Yes	67	7.64	100
Time spent on ART, years			
<2	79	9.01	9.01
2-<4	141	16.08	25.09
4-<6	273	31.13	56.21
6-9	384	43.79	100

**Table 3:** Description of numerical variables (n=877) BMI: body mass index; SD: standard deviation

Summary of numerical variables			
	Age, years	Time spent on ART, years	BMI, kg/m^2^
Stats			
Minimum	18	0.2	12.98476
SD	11.03781	2.11237	4.549935
Mean	40.35918	5.331243	24.32783
Lower quartile (Q1)	33	3.9	21.48438
Median (Q2)	40	5.6	23.87511
Upper quartile (Q3)	47	6.9	26.80696
Maximum	81	9	56.5547

Among the 877 patients with full information on hypertension status and time lived on ART, the majority were from the Masvingo district (33.3%) followed by the Chiredzi district (26.45%), with the least number of patients hailing from the Mwenezi district (4.1%). The minimum age among the patients in the study was 18 years while the maximum was 81 years. The interquartile range (IQR) for age was 14 with the age distribution symmetric with the mean median and mode all being 40 years of age. The majority of the patients were in the age group 36-45 years (37.4%) followed by those in the 26-35-year age group (25.31%), while the least number was in the age group above 65 years (2.96%).

There were more females (63.17%) than males (36.83%) in the analyzed sample. The majority of the clients were married (65.68%) followed by those that are divorced (14.37%) and those that were widowed (10.49%), and only 9.46% of the patients were single. The BMI of the patients was classified as follows: <18.5: underweight; between 18.5 and <25: normal; between 15 and <30: overweight; 30 and above: obese.

The majority of patients had a normal BMI (50.5%), with 29.99% being overweight, 8.67% being obese, and 6.84% being underweight. The duration of ART ranged from 0.2 years to nine years in the patients analyzed with a mean of 5.3 years, median of 5.6 years, and the mode being with an interquartile range of three years. Most of the patients in the study had spent six to nine years (43.79%) on ART, followed by those who had spent 4-<6 years (31.13%) and those who had spent from 2 up to <4 years (16.08%); only 9.01% had spent <2 years on ART. The prevalence of hypertension among people living with HIV and on ART in this sample was 7.64% (Table 2). Table 4 summarizes the clinical and demographic characteristics based on the hypertension status of the study participants.

**Table 4:** Clinical and demographic characteristics based on the hypertension status ART: antiretroviral therapy: BMI: body mass index

Variable	Hypertensive	
Covariate	No, n (%)	Yes, n (%)
District		
Chiredzi	204 (87.93%)	28 (2.07%)
Chivi	169 (99.41%)	1 (0.59%)
Gutu	79 (91.86%)	7 (8.14%)
Masvingo	264 (90.41%)	28 (9.59%)
Mwenezi	35 (97.22%)	1 (2.78%)
Zaka	59 (96.72%)	2 (3.28%)
Age group, years		
18-25	74 (98.67%)	1 (1.33%)
26-35	217 (97.75%)	5 (2.25%)
36-45	311 (94.82%)	17 (5.18%)
46-55	158 (85.87%)	26 (14.13%)
56-64	32 (76.19%)	10 (23.81%)
65+	18 (69.23%)	8 (30.77%)
Gender		
Female	515 (92.96%)	39 (7.04%)
Male	295 (91.33%)	28 (8.67%)
Weight/BMI		
Underweight	59 (98.33%)	1 (1.67%)
Healthy weight	449 (93.93%)	29 (6.07%)
Overweight	240 (91.25%)	23 (8.75%)
Obese	62 (81.58%)	14 (18.42%)
Marital status		
Divorced	117 (92.86%)	9 (7.14%)
Married	531 (92.19%)	45 (7.81%)
Single	80 (96.39%)	3 (3.61%)
Widowed	82 (89.13%)	10 (10.87%)
Time spent on ART, years		
<2	78 (98.73%)	1 (1.27%)
2-4	139 (98.58%)	2 (1.42%)
5-6	247 (90.48 %)	26 (9.52%)
7-9	346 (90.10%)	38 (9.90%)

The Masvingo and Gutu districts had the highest prevalence of hypertension at 9.59% and 8.14% respectively compared to other provinces. The prevalence of hypertension increased with age; the prevalence was higher in patients aged >45 years and the highest prevalence was observed in the age group of >65 (30.77%). BMI was found to be associated with a higher risk of hypertension and the observed prevalence was three times higher in obese patients compared to those with normal weight. The prevalence of hypertension was 18.2% in obese patients compared to 6.2% in patients with normal weight. Moreover, there was an increased prevalence of hypertension in patients who had spent more time on ART. The prevalence was found to be 9.52% and 9.90% in patients on ART for five to six and seven to nine years respectively.

Logistic regression

Before the forward selection method on the initial regression model in which all variables with p<0.25 were retained, in the final multivariate regression model, only marital status was dropped while sex, age, BMI, and district were retained. The researchers also included time spent on ART in the final model as it was one of the variables of interest for the topic.

**Table 5:** Multiple logistic regression of hypertension risk factors ART: antiretroviral therapy: BMI: body mass index

Logistic regression				Variable	Value	
				LR chi2 (5)	69.33	
				Prob > chi2 =	0.0000	
Log-likelihood	-202.02103			Pseudo R2 =	0.1465	
Hypertensive	Odds ratio	Standard error	z	P>|z|	95% confidence interval	
Age	1.080506	0.0130749	6.40	0.000	1.055182	1.106439
BMI	1.088751	0.027824	3.33	0.001	1.03556	1.144674
Sex	1.114179	0.331955	0.36	0.717	0.6213705	1.997833
Time spent on ART	1.215188	0.0938327	2.52	0.012	1.04452	1.413743
District	1.091679	0.1004225	0.95	0.340	0.9115779	1.307363
Cons	0.000067	0.0000812	-7.92	0.000	6.21E-06	0.0007223

The final multivariate logistic regression model identified age as a highly significant risk factor for hypertension among people on ART (OR: 1.08, 95% CI: 1.055-1.11, p=0.000). The risk of hypertension increases by approximately 8% with a one-year increase in age. BMI (OR: 1.088, 95% CI:1.036-1.45, p=0.001), and an increase in BMI by a factor of 1 increasing the risk of getting hypertension by 9%. Time spent on ART treatment was also found to be significantly associated with hypertension (OR: 1.21, 95% CI: 1.04-1.41, p=0.12). This means that an increase in one year spent on ART was found to be associated with a 21% increase in the risk of hypertension. Sex and district were found not to be significantly associated with hypertension after controlling for other covariates in the model.

## Discussion

The prevalence of hypertension among ALWHIV is not well documented but believed to be higher than among those who are not infected by HIV because of the effects of HIV infection and the adverse effects of ART [13]. A review of literature from sub-Saharan Africa in 2018 showed that the estimated pooled prevalence of hypertension among ALWHIV was 21.2% (95% CI: 16.3-27.1) [5]. Hypertension has been identified as an important risk factor for cardiovascular diseases and an increase in hypertension increased the risk of acute myocardial infarction by 20% [14,15]. A study conducted in Maryland showed that the prevalence of hypertension was highest (23%) among those who had perinatally acquired HIV compared to those with non-perinatally acquired HIV (10%) and HIV-uninfected people (8%) [16]. In a study conducted in South African public hospitals, the prevalence of hypertension among ALWHIV was 38.6% (95% CI: 34.3-42.9) and 46.3% of those with hypertension were aware of their hypertension status [17]. Another investigation of HIV comorbidity with hypertension and diabetes in Ethiopia estimated the prevalence of hypertension at 14.1% [18].

In Zimbabwe, there have been only a few studies to estimate the prevalence of hypertension or NCDs among HIV-infected adults resulting in a paucity of data on the actual burden of NCDs and HIV comorbidity, similar to many other low to medium-income countries [5,19]. A study to determine the prevalence of hypertension among ALWHIV at a central hospital in Harare revealed that the prevalence of hypertension was 29.9%; of these, 11.2% were not aware of their hypertensive status before the screening conducted during the study [19]. The prevalence of hypertension was higher in males (31.6%) than in females (28.1%) though the finding was not statistically significant. In a study conducted in Uganda, it was found that being HIV infected was not associated with hypertension because the HIV-infected participants were less likely to have the traditional risk factors for hypertension such as obesity and overweight (BMI of 25-30 kg/m²) [20]. However, the paucity of data and evidence on this in sub-Saharan Africa may be responsible for missed opportunities in diagnosis and quality care of hypertension in HIV care [21].

A literature review on hypertension in HIV-infected adults by Fahme et al. demonstrated that ART may mediate hypertension in ALWHIV; for instance, protease Inhibitors (PIs) can result in peripheral and central adiposity accumulation and lipodystrophy linked to hypertension [13]. Lipoatrophy and lipodystrophy are related to imbalances in intracellular cholesterol, and they independently predict hypertension in ALWHIV. Contrary to these findings, a study in Harare found no association between PI and non-PI-based ART regimens in the prevalence of hypertension among ALWHIV [19]. High proportions of undiagnosed hypertension have been reported among both HIV-infected and HIV-uninfected adults [7,15,18,19,20,22].

This study aimed to determine the burden of hypertension in HIV-infected clients on ART. The prevalence of hypertension in people on ART is comparable to the overall prevalence of hypertension among the general population in Zimbabwe [4]. The results from this study show an increase in the risk of hypertension with an increase in years spent on ART. However, in this study, the prevalence of hypertension was lower than that found in other studies in Zimbabwe among ART patients, and this could be due to a number of factors such as poor completion of records resulting in fewer records being analyzed. It should be noted our findings could still reflect the actual prevalence in the Masvingo setting as there has not been any previous study in that area and also because there have been few national studies to determine the prevalence of hypertension in HIV clients on ART [4,19]. This contradicts the perception from study findings that chronic ART intake is a risk factor for hypertension.

An increase in age was found to be a risk factor for hypertension among people on ART treatment. The steady increase observed in the prevalence of hypertension in those who are 65 years and above, i.e., 30.77% from a prevalence of 1.33% in those 18-25 years of age, is consistent with the findings from studies in other parts of the world: a study from the United States has reported that the prevalence of hypertension is 7% in those 18-39 years of age and rises to about 65% in those who are 60 years old and above [23]. A study from Uganda has noted that increasing age and ART were significantly associated with hypertension with higher mean values for both systolic and diastolic blood pressure [24].

It is well known that the socioeconomic situation in Zimbabwe has also resulted in a significant brain drain in the health sector due to experienced nurses leaving the country in large numbers, thereby resulting in a shortage of nurses in the health facilities with a consequent increase in workload. This results in poor documentation, primarily due to the lack of experience among the available staff as well as the pressure of work as, in the public health system, the primary source documents are the patient files and not the ePMS system, and most nurses and clinicians hold the belief that this represents extra work. This situation has been worsened by the recent introduction of the Electronic Health Records system, which is further aggravating the workload. It is a separate system that the government is introducing with a view to improving patient monitoring and clinical efficiency. The ePMS system does not function properly all the time and depends on the availability of trained staff, electricity, and functional laptops; thus, the information available is frequently complete and there have been instances of data being not captured for long periods, resulting in a need for back-capturing of data, which makes it difficult to have the accurate and correct information.

Studies on ART patients noting their cardiometabolic risk have reported an increased risk of hypertension with an increase in the duration of ART. This has been attributed to the elevation of metabolic agents such as total cholesterol, low-density lipoproteins, and high-density lipoproteins. However, it has also been noted that certain antiretrovirals like PIs reduce the risk of hypertension and more time spent on these would also reduce the risk of developing hypertension, which contrasts with some other studies reporting that being on PIs increases lipodystrophy which would, in turn, increase the risk of hypertension. Because patients can be on different medications, due to factors such as availability, patient toxicities, or even for the purpose of controlling control viremia, it is important that clinicians know what medications their clients are on as they may increase the risk of hypertension as well as cardiovascular risk factors.

Several studies have found that there is a significant difference in the prevalence of hypertension between males and females with males having a higher prevalence than females overall; however, in this study, there was no significant difference between the prevalence of hypertension between males and females. In a study conducted at Parirenyatwa Hospital in Zimbabwe, there was a higher prevalence of hypertension in males (31.6%) than in females (28.1%) though the difference was not statistically significant [19,25,26].

Obesity is a recognized independent risk factor for the development of hypertension and cardiovascular disease, and this has been reinforced in our study where, on univariate and multivariate analyses, obesity remained a significant risk factor for hypertension [25]. Obesity, especially abdominal obesity, can lead to the development of metabolic syndrome, which also increases the risk of hypertension and cardiovascular disease as the abdominal adiposity is associated with hormonal secretion, which produces hormones that promote hypertension and an increase in pre-diabetes and diabetes due to increased insulin resistance [27].

An increase in BMI was also found to be associated with an increased risk of hypertension among people on ART treatment. Significantly, upon a separate analysis, an increase in BMI from lower to higher categories was also found to be associated with an increase in years lived on ART, especially among patients with a good adherence history. This is a point worth noting because overweight and obesity are known risk factors for hypertension. Thus, if chronic ART intake is associated with a BMI category increase, it follows that chronic ART intake is also indirectly associated with hypertension. This could be crucial for hypertension prevention and management among ART patients, especially with regard to program planning and implementation [26].

Strengths

The study adds to the body of knowledge on hypertension in HIV care. The sample size of 877 was large enough to produce valid results and thus the findings of this study can be used to devise policies on HIV care and management and improve the quality of care of patients in the public healthcare system.

Limitations

Some biases may have crept in due to the retrospective nature of the study design. An effort to reduce some of the biases was made by excluding patients with incomplete demographic, treatment, and clinical data or any other data that we deemed vital for the study. We also excluded patients who were no longer receiving active ART. A small number of patients with missing records were not included in the study. The other major limitation pertains to the data availability in the ePMS database. There are very few variables recorded in the surveillance system, and hence we could not assess some of the factors that have been hypothesized by other researchers to be associated with hypertension among people actively on ART.

Recommendations

This research project identified the prevalence of hypertension among the patients related to certain covariates such as age, duration on ART, and BMI; however, we could not analyze certain other variables due to the unavailability of the data. Hence, a further study would be required to look into these other variables to ensure a holistic approach to the management of risk factors for hypertension in HIV care. More studies involving the analysis and measurement of the incidence of hypertension are required as these will aid with meaningful policy analysis and review.

There is a need for data quality standard operating procedures to ensure that all critical variables that affect the quality of care in HIV clients are captured and accounted for. This should be followed up by the set-up of data quality indicators to monitor the data-capturing process. HIV care in Zimbabwe includes a feedback loop with the clients themselves that assesses the care that they are receiving; based on this data, while the researcher thinks that even though clients have rights and that they should advocate for improved care, they are simply unaware that they need to have NCD checks regularly as they also affect their quality of life [16,18]. A significant percentage of the population in Zimbabwe does not believe in exercise and diet, and yet evidence has shown that this works to reduce the burden of NCDs including hypertension [4,22]. As behavioral change requires empowerment strategies, there is a need for investment to ensure that there are enough health promotors and dieticians in the communities Who can help with this behavior change initiatives, as these do not need to be all talk but can be practical by using the available resources in the respective communities for it to be successful. There is a need to continuously monitor the quality of care for HIV clients on the newer regimens of ART as some effects may be salient. As most of the resources in HIV care are being provided by USAID and other partners, they need to be approached to discuss the need to fund the NCD program as it is currently suffering from a lack of resources. This research has shown that need as HIV clients may do well on ART but may continue to burden the healthcare system due to NCDs that are poorly managed, and this can increase their morbidity and mortality [28,29].

Public health implications of the study

The findings from this study can be used to improve the management of HIV-related NCDs in Zimbabwe and throughout the sub-Saharan Africa region, which bears the major brunt of the HIV pandemic [1,3,16]. Patients on ART face an increased risk of NCDs with increasing age, due to the effect of antiretroviral medications and the HIV virus itself, and a host of lifestyle effects such that there is a need to focus on them to improve the quality of life with reduced morbidity and reduction in mortality. Hence, the public health system needs to remove the silo mentality in terms of managing NCDs and HIV separately and manage them together, and this requires a shift in the mindsets of the clinicians who interface with patients as well the policymakers that create the silo mentality [4,30]. There is a need to integrate programming to be inclusive of both of these issues so that resources are used effectively and, most importantly, the patients receive the quality of care that they deserve. Overall, tackling the HIV pandemic along with NCDs needs a mindset shift among policymakers as this has huge implications on resources and not acting has dire consequences, while proper planning that includes prevention strategies may in the long run lead to reduced costs that are incurred by the public healthcare system. Even though ART can be lifesaving, there is a need to continuously monitor patients on ART for cardiovascular risk factors including those related to hypertension.

## Conclusions

The study demonstrated that there is a significant prevalence of hypertension in HIV-positive patients on ART and factors of age and obesity are independent risk factors along with the duration of ART. The secondary data analysis showed that there are gaps in the data collected by the facilities and could have hindered the assessment of overall prevalence rates; however, the study did present important outcomes while also highlighting that more needs to be done on data quality management as well as on improving the quality of HIV care as data on some important variables are not being collected. Lifestyle interventions to manage NCD prevalence need to be supported with the provision of resources as they are critical for the improved care of HIV patients.
